# Molecular Pathways and Targeted Therapies in Relapsed/Refractory Diffuse Large B-Cell Lymphoma (DLBCL)

**DOI:** 10.3390/cancers17142314

**Published:** 2025-07-11

**Authors:** Jonathan Weiss, Shannon A. Carty, Yasmin H. Karimi

**Affiliations:** 1 Division of Hematology-Oncology, Department of Medicine, University of Michigan, Ann Arbor, MI 48109, USA; scarty@med.umich.edu (S.A.C.); karimiy@med.umich.edu (Y.H.K.); 2 Rogel Cancer Center, University of Michigan, Ann Arbor, MI 48109, USA

**Keywords:** signaling, non-Hodgkins, immunotherapy, targeted therapy, BCR, TLR, CD19, ROR1, CD30, PD-1, XPO-1

## Abstract

There have been multiple approved agents for relapsed/refractory (r/r) Diffuse Large B-cell Lymphoma (DLBCL) over the last 8 years. The majority of these therapies act on specific signaling pathways in malignant B-cells. These signaling pathways stem from the B-cell receptor (BCR), Toll-Like Receptor (TLR), PI3K/AKT/mTOR, BCL-2, and XPO-1. In addition, novel therapies that target extracellular proteins (CD19, CD20, CD30, ROR1, and PD-1) have been developed. The purpose of this review is to discuss the various therapies that target these pathways and highlight the success and shortcomings of these novel agents.

## Simple Summary

Over the last 8 years, there have been multiple new therapies approved for relapsed/refractory (r/r) Diffuse Large B-cell Lymphoma (DLBCL). These therapeutics act on specific signaling pathways in malignant B-cells. This is a review article that details the molecular signaling pathways in DLBCL as well as the novel agents that target these pathways.

## 1. Introduction

**Figure 1:**
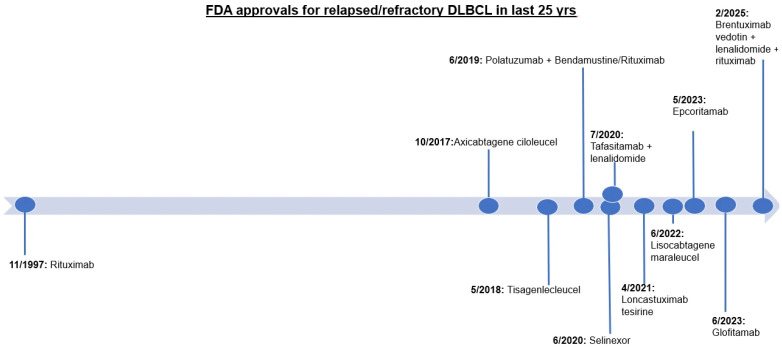
A timeline of FDA approvals for relapsed/refractory DLBCL over the last 25 years.

Diffuse Large B-Cell Lymphoma (DLBCL) is the most common type of lymphoma in the world, accounting for approximately 30% of all cases of Non-Hodgkin’s lymphoma (NHL) and more than 20,000 annual cases in the US [1]. In regard to clinical presentation, most patients present with an enlarging mass occurring in the lymph nodes, though DLBCL can arise anywhere on the body. Frequently, DLBCL is associated with B symptoms (i.e., fever, weight loss, and drenching night sweats). The diagnosis is made through tissue biopsy, with pathologic review including morphology, immunophenotype, and cytogenetic features. DLBCL is heterogenous in regard to its pathologic profile, with multiple different classifications based on the cell of origin, genetic/molecular subtypes, and morphologic variants. R-CHOP (rituximab, cyclophosphamide, doxorubicin, vincristine, and prednisone) was approved by the FDA in 2006 and has been the mainstay of front-line therapy for the last two decades. In 2023, Polatuzumab vedotin, rituximab, cyclophosphamide, doxorubicin, and prednisone (i.e., Pola-R-CHP) gained FDA approval after it was found to improve progression-free survival (PFS) relative to R-CHOP, and remains an acceptable front-line treatment strategy as well [2]. These strategies (R-CHOP or Pola-R-CHP) alone are able to cure approximately 60–70 percent of patients [3]. However, the remaining patients experience relapsed/refractory disease. Prior to 2017, options in the relapsed/refractory setting were limited to immuno-chemotherapy with or without autologous stem cell transplant consolidation. Over the last 8 years, the FDA has granted accelerated approval to multiple new agents in relapsed/refractory DLBCL including polatuzumab in combination with rituximab-bendamustine [4], selinexor [5], tafasitamab with lenalidomide [6], loncastuximab tesirine-lpyl [7], brentuximab vedotin with lenalidomide + rituximab [8], and multiple CD19 chimeric antigen receptor (CAR) T-cell therapy options and bispecific antibodies (BsAbs) [9,10,11,12,13] (Figure 1). Despite these novel therapies, select patients with relapsed/refractory DLBCL continue to have poor outcomes. A continued understanding of tumor biology and signaling pathways will help pave the way for novel therapies in DLBCL and improve patient outcomes. The purpose of this review is to provide an overview of molecular signaling in DLBCL and detail the therapeutic targets that disrupt these pathways.

## 2. Classifying DLBCL

There have been multiple efforts to classify and group DLBCL based on pathologic and molecular characteristics, including cells of origin, genetic/molecular subtypes, immunophenotypes, and cytogenetic features. Gene expression profiling has identified subgroups of DLBCL according to cell of origin. DLBCL can arise from either a germinal center B-cell (GCB) or a post-germinal center activated B-cell (non-GCB). Determining the cell of origin is important for prognostication and management [14,15]. The GCB subtype has a more favorable five-year survival rate of 76% with standard R-CHOP when compared to the ABC subtype with a five-year OS of 56% [16]. Cell of origin classification with gene expression profiling techniques are both cumbersome as well as expensive and are not generally pursued in clinical practice. Instead, immunohistochemistry algorithms (e.g., the Hans algorithm) evaluating MUM-1, CD10, and BCL-6 protein expressions are more commonly used to determine the cell of origin [17]. The most commonly used approach is the Hans algorithm, which is able to distinguish GCB-subtype large-cell lymphomas from non-GCB subtypes with a 79% correlation for COO using the Hans algorithm compared to gold standard gene expression profiling.

More recently, DLBCL has been further classified beyond the cell of origin to distinct subsets or “clusters” using recurrent mutations, somatic copy number alterations, and structural variants (e.g., MCD, N1, BN2, EZB, and other GCBs). These subgroups have been found to be of clinical importance when considering prognostics and in which patients are expected to have inferior prognosis with standard immuno-chemotherapy [18,19].

Cytogenetics has also played a role in classifying DLBCL. Patients with MYC and BCL2 and/or BCL6 genetic rearrangements have poor outcomes and a designation of High-Grade B-cell Lymphoma (HGBCL). These lymphomas are also termed “Double/Triple Hit Lymphoma.” Retrospective evidence supports intensive regimens (i.e., DA-EPOCH-R) in these high-risk lymphomas [20].

Depending on the cell of origin and DLBCL molecular subtype, different signaling pathways are altered and, therefore, offer different potential therapeutic targets. For example, the ABC subtype has a “chronically active” B-cell receptor (BCR) signaling pathway with constitutive activity of nuclear factor kappa beta (NF-κβ) activity [21]. The GCB subtype, on the other hand, has a “tonic”, antigen-independent BCR signaling pathway, leading to AKT activation [22]. As a result, therapeutics that target the “chronic active” BCR pathway are efficacious in ABC DLBCL [23]. The BCR signaling pathway, as well as other relevant pathways and their therapeutic targets, are described in more detail throughout this review.

## 3. Molecular Pathway Targets

### 3.1. BCR Signaling in DLBCL

**Figure 2:**
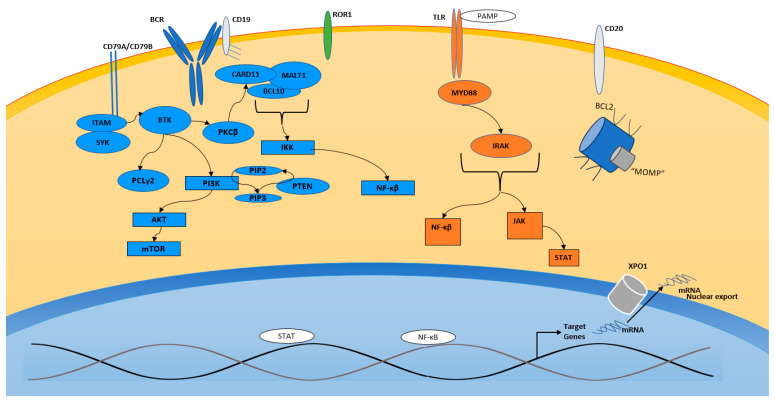
Illustration of multiple B-cell signaling pathways and extracellular targets, including B-cell receptor (BCR), Toll-Like Receptor (TLR), CD19, CD20, XPO1, and mitochondrial “MOMP” pathway.

The BCR is paramount for B-cell development and differentiation. Structurally, the BCR consists of a pair of membrane-bound antibodies coupled to CD79A and CD79B. In a non-malignant B-cell, signaling is initiated by the recognition of an antigen by the BCR, which results in a signaling cascade via the phosphorylation of CD79A and CD79B [24]. Following signal initiation, CD79A and CD79B trigger spleen tyrosine kinase (SYK), which ultimately leads to the activation of Bruton tyrosine kinase (BTK) [25]. BTK then activates multiple downstream proteins. This protein cascade results in the translocation of CARD11 to the inner side of the plasma membrane. CARD11 is associated with BCL10 and mucosa-associated lymphoid tissue (MALT1) and forms the CBM complex. CBM triggers Iκβ kinase (IKK) and the NF-κβ pathway [21,26]. (illustrated in Figure 2). NF-κβ refers to a group of proteins that function as transcription factors and are important for cell proliferation and survival.

BCR signaling in DLBCL can be classified as either “chronic active” or “tonic” BCR signaling. In general, the ABC subtype exhibits a “chronic active” BCR signaling pathway, while GCB follows a “tonic” BCR signaling pathway. “Chronic active” BCR signaling results in the clustering of BCR and BCR-dependent NF-KB activation. ABC DLBCL expresses NF-κB-dependent genes and relies on NF-κβ for survival. Mutations in CARD11, CBM complex, ITAM, or SYK can give rise to “chronic active” BCR signaling and the activation of NF-κB, which is seen in the ABC subtype DLBCL [21,22,27]. In comparison, the GCB subtype DLBCL follows a “tonic” BCR signaling pathway that is antigen-independent, mediated by PI3k/AKT/mTOR pathway, and lacks NF-κB pathway activity [28]. NFAT is a transcription factor similar to NF-κB. The inhibition of NFAT may have therapeutic implications for the ABC subtype DLBCL [29].

Therapies that target the BCR-dependent pathway have been evaluated, and these include inhibitors of BTK, SYK, PKCB, and MALT1. The BTK inhibitor ibrutinib has been evaluated as a single agent in relapsed/refractory DLBCL. The overall response rates were suboptimal in 20/80 (25%) of the treated patients. However, there was a dramatic difference between ABC and GCB subtype DLBCL. When broken down by gene expression profiling, the ABC subtype had an overall response rate in 14/38 patients (37%), whereas the GCB subtype had responses in only 1/20 patients (5%). This supports the notion that chronic active BCR signaling confers sensitivity to ibrutinib, while tonic BCR signaling does not respond as well [23]. SYK inhibition has also been evaluated in DLBCL with overall discouraging results. The SYK inhibitor fostamatinib was studied in relapsed/refractory DLBCL with an ORR of 3% [30]. Similarly, the SYK inhibitor entospletinib was evaluated with 43 patients with relapsed/refractory DLBCL and had no responses [31]. The inhibition of PKCβ was studied with enzaustaurin in patients with relapsed/refractory DLBCL. Of the 55 patients enrolled, 12/55 patients experienced freedom from progression over two 28-day cycles, and 8/55 patients experienced freedom from progression for four or more cycles [32]. Targeting BCR pathways continues to be an active area of exploration. For example, small molecules that target MALT1 have been found to suppress ABC-DLBCL in vitro [33] and are currently being evaluated in phase 2 clinical trials in combination with ibrutinib (clinicaltrials.gov, NCT04876092).

### 3.2. Toll-Like Receptor (TLR) Pathways in DLBCL

TLRs also play a role in B-cell activation, and aberrant TLR signaling has been associated with malignancies. In terms of structure, TLRs have an extracellular domain and a transmembrane and intracellular toll/interleukin 1 domain or “TIR” [34]. When activated, the TIR domain can recruit myeloid differentiation primary-response protein 88 (MYD88) [35]. MYD88 plays a recruitment role for IL-1R-associated kinases (IRAK), and this results in the nuclear translocation of NF-κβ. The most common mutation is MYD88L265P, which occurs exclusively in ABC DLBCL and is associated with extranodal DLBCL involvement and poor outcomes [36]. When mutated, MYD88 results in the overactivation of both NF-κβ and JAK-STAT signaling (illustrated in Figure 2). The constitutive activation of NF-κβ is associated with the ABC subtype DLBCL and ultimately results in poor outcomes [28].

Preclinical studies have shown that targeting TLR and IRAK kinases has resulted in an anti-tumor effect in DLBCL [37,38]. Attempts have also been made to target NF-κβ. Lenalidomide is one such therapy that inhibits NF-κβ activity in non-GCB DLBCL cell lines [39]. Single-agent lenalidomide has been used in relapsed/refractory DLBCL with an overall response rate of 28% [40]. More recently, combinations of lenalidomide with B-cell targeting monoclonal and bispecific antibodies have shown more efficacy, and these are discussed in more detail later in this review. Golcadomide is a novel cereblon E3 Ligase modulator (CELMoD) that works in a similar fashion to lenalidomide by inducing the degradation of ikaros/aiolos, which are transcription factors necessary for B-cell malignancy development. The ORR with golcadomide was 50% in a high-risk patient population [41].

### 3.3. PI3K-AKT-MTOR in DLBCL

The phosphoinositide 3-kinase (PI3K)/protein kinase B (AKT)/mammalian target of the rapamycin (mTOR) cellular signaling pathway is important for cellular proliferation, metabolism, and survival, and it has been found to be dysregulated in non-Hodgkins lymphoma [42]. The main function of PI3K is to phosphorylate phosphatidylinositol (4,5) bisphosphate (PIP2) to generate phosphatidylinositol (3,4,5)-triphosphate (PIP3), which is a secondary messenger and activates multiple downstream targets of the PI3K pathway [43]. The phosphatase and tensin homolog (PTEN) converts PIP3 into an inactive substrate, PIP2 [44]. When altered, PTEN can be associated with malignancy due to its inability to convert PIP3 into inactive substrate, which results in cellular proliferation [44,45,46]. PI3K/AKT/mTOR pathway inhibition has been studiedin DLBCL. Patients with r/r DLBCL were treated with the PI3K inhibitor copanlisib. The ORR was 19.4%, with higher rates of response in the ABC subtype versus the GCB subtype [47]. Similarly, idelalisib, a PI3K inhibitor, showed an ORR of 14%, with slightly higher ORRs for the ABC subtype than the GCB subtype. The AKT inhibitor MK2206 was evaluated in relapsed/refractory DLBCL with overall dismal response rates. mTOR has also been studiedas a potential target in relapsed/refractory DLBCL; in a phase 2 study of everolimus with 24 patients, the overall response rate was 38% [48].

### 3.4. BCL2 in DLBCL

Apoptosis activation can occur through mitochondrial outer membrane permeability (MOMP) formation. This process leads to the release of multiple pro-apoptotic molecules and subsequent apoptosis [49]. The permeability of the mitochondria membrane is controlled by the BCL-2 family. The balance between pro-apoptotic and anti-apoptotic proteins can lead to the disruption of the cell-dying process and the ultimate survival of cancer cells [50]. In DLBCL, BCL2 overexpression resulted in apoptosis inhibition and maintained tumor viability [51]. BCL2 inhibition with venetoclax was evaluated in relapsed/refractory DLBCL with an unimpressive overall response rate of 18% [52]. While single-agent venetoclax produces unsatisfactory results, there have been strategies to combine BCL-2 inhibitors with other targeted therapies with favorable results. For example, the ViPOR regimen, which includes venetoclax, ibrutinib, prednisone, obinutuzumab, and lenalidomide, had an ORR of 54% and CRR of 38% in a high-risk patient population with r/r DLBCL in which some patients had durable responses [53].

### 3.5. XPO1

The transport of larger molecules from the nucleus to the cytoplasm requires various transport receptors, including exportin 1 (XPO1). XPO1 is important for the nuclear export of multiple tumor suppressor proteins, including p53, and is overexpressed in DLBCL [54,55]. Oncoprotein mRNA is exported out of the nucleus by XPO1, facilitating the cytoplasmic translation and increasing levels of oncoproteins [56]. Selinexor is an oral selective inhibitor of XPO1-mediated nuclear export. The SADAL trial was a multicenter open-label phase 2b study evaluating selinexor in patients with relapsed/refractory DLBCL. The overall response rate was 28% (36/127), with 12% of patients (15/127) receiving a complete response [5]. The side effect profile of selinexor is noteworthy and includes fatigue, nausea, pancytopenia, and pyrexia. Given the poor overall response rates and side effect profile, selinexor is not frequently used in clinical practice despite FDA approval.

## 4. Surface Receptor Targets

The first extracellular target in DLBCL was against the B-lymphocyte antigen CD20 with the monoclonal antibody rituximab. Several studies in B-NHL, including DLBCL, have shown impressive progression-free and overall survival improvements when rituximab is incorporated with standard chemotherapy backbones. Since 2006, rituximab plus chemotherapy has been the mainstay first-line therapy in DLBCL. Additional CD20 targets have included obinutuzumab, as well as multiple BsAbs. BsAbs retarget T cells to a specific antigen on tumor cells. There are now two FDA-approved CD20xCD3 bispecific antibodies for the treatment of relapsed/refractory DLBCL—glofitamab and epcoritamab. Glofitamab has been shown to be effective in relapsed/refractory DLBCL with an ORR of 50% and a CR rate of 35% [9]. Epcoritamab has an overall response rate of 59%. The median PFS was 4.2 months; however, among patients with a CR, PFS was 37.3 months [13,57]. The STARGLO trial combined Glofitamab with gemcitabine and oxaliplatin in second-line and beyond relapsed/refractory DLBCL and found an overall survival benefit to the combination compared to gemcitabine and oxaliplatin alone [58]. While not approved for DLBCL, mosunetuzumab is another CD20/CD3 bispecific antibody that is being evaluated in patients with R/R DLBCL in combination with polatuzamab-vedotin and has response rates of 59.2% [59]. Similarly, odronextamab has been evaluated in r/r DLBCL (though not yet approved) with an ORR of 52% and CRR of 31% [60].

CD19 is a B-cell coreceptor that plays a role in B-cell activation and development. It is a transmembrane protein with three cytoplasmic tyrosine residues, which facilitate the majority of biologic functions. When activated, it can amplify the activation of the PI3k/AKT/mTOR pathway [61]. The anti-CD19 target tafasitamab was used in combination with lenalidomide in a multicenter, open-label, phase 2 study (L-MIND), including patients with relapsed/refractory DLBCL [6]. The results were overall encouraging, with an ORR of 48/80 (60%), which led to FDA approval. More recently, real-world outcomes have shown less promising ORRs than those seen in the L-MIND trial at only 31% and a median PFS of 1.9 months [62]. Loncastuximab tesirine is an antibody directed towards CD19 and is conjugated to an alkylating agent. Lonastuximab tesirine was evaluated in the phase 2 trial (LOTIS-2) in patients with relapsed/refractory DLBCL with an ORR of 70/145 (48.3%) and a CR rate of 35/145 (24.1%) [7]. Additional CD19 targets include bispecific antibodies. For example, blinatumomab, a CD19xCD3 bispecific antibody, was studied in the relapsed/refractory setting with an ORR of 37% and CR rate of 22% [63].

In addition, there are three FDA-approved CAR-T cell therapies that can be used in the third-line setting that target CD19. These agents include axicabtagene ciloleucel, tisagenlecleucel, and lisocabtagene maraleucel. These therapies all showed promising results in the third-line setting [11,12,64]. Axicabtagene was evaluated in a phase 3 trial in the second-line setting in those patients with primary refractory disease or early relapse (defined as less than 12 months). The results showed an ORR of 83% and improved PFS and OS compared to the standard of care (i.e., chemo-immunotherapy plus autologous stem cell transplant) [65]. Similarly, lisocabtagene maraleucel was evaluated in a phase 3 clinical trial in patients with DLBCL with relapse experienced less than 12 months after initial therapy or in patients with primary refractory disease and improvements in PFS [66]. Tisagenlecleucel was evaluated in the second line as well, with no PFS or OS benefit over the standard of care.

As detailed previously, the BCR is coupled to CD79A and CD79B. Polatuzumab vedotin is an antibody–drug conjugate composed of an anti-CD79b monoclonal antibody conjugated to a microtubule inhibitor. Polatuzumab was evaluated in combination with bendamustine and rituximab (pola + BR) in relapsed/refractory cases. There was a significant overall survival benefit with pola + BR relative to the BR cohort (12.4 vs. 4.7 months) [2].

An additional cell surface target is the receptor tyrosine kinase-like orphan receptor 1 (ROR1). ROR1 drives the physiology of embryonic stem cell proliferation but is also found to be present in hematologic malignancies [67]. The antibody–drug conjugate zilovertamab vedotin, which is an ROR-1 target, has been studied in patients with r/r DLBCL. Response rates among the 79 evaluable patients were 23/79 (29%), with 10 complete responses and 13 partial responses [68].

CD30, an activation-induced antigen expressed in subsets of normal T-cells and B-cells, is expressed in DLBCL approx. 30% of the time and is another potential therapeutic target [69]. Brentuximab-vedotin (BV) is an antibody–drug conjugate that targets CD30 and is currently approved for use in anaplastic large-cell lymphoma and classical Hodgkin lymphoma. BV has also been evaluated in CD30 + DLBCL with ORRs of 44% and a median duration of response of 16.6 months [70]. BV was combined with lenalidomide in a phase 2 study with an ORR of 57%, with 35% of patients achieving a CR [71]. The FDA recently approved BV in combination with rituximab and lenolidomide for r/r DLBCL, with an improvement in overall survival of 13.8 months compared to 8.5 months with rituimxab/lenolidomide [8]. It is worth noting that CD30 expression does not seem to correlate with response—patients who are CD30-negative consistently respond to BV.

## 5. Checkpoint Inhibitors

Program death-1 (PD-1) and its ligands PD-L1/PD-L2 are important for preventing autoimmunity and maintaining self-tolerance [72]. In patients with r/r primary mediastinal B-cell lymphoma (PMBCL) who received pembrolizumab, the ORR was 45% (25/53) with a CR rate of 13% (7/53) [73] Similarly, BV + Nivolumab was studied in a phase 1/2 open-label multicenter study in patients with r/r PMBCL and determined an ORR of 73.3% and median PFS of 26.0 months [74].

PD-L1 gene alterations impact the response to checkpoint blockade. Through the analysis of 105 DLBCL cases, it was illustrated that PD-L1 alterations are more common among the non-GCB subtype DLBCL and have robust PD-L1 protein expressions. Patients with PD-L1 alterations have an inferior PFS with front-line therapy. However, they do demonstrate improved response to anti-PD-1 therapy and may identify a subset of DLBCL that is more responsive to checkpoint blockade [75].

The PD-1 blockade with nivolumab produced unsatisfactory results when studied in relapsed/refractory DLBCL. The overall response rate was 3/87 (3%) among patients who had previously failed autologous transplants [76]. Overall, targeting PD-L1 outside PMBCL has been ineffective.

CD47 is an additional immune checkpoint in which a blockade seems to provide more promising results in patients with relapsed/refractory DLBCL. CD47, also known as the “don't eat me signal”, prevents tumor cell phagocytosis, promotes tumor progression, and allows cancer cells to avoid immune surveillance. When overexpressed in DLBCL, it portends a poor prognosis [77]. The blockade of CD47 has been evaluated in a phase 1b study in patients with relapsed/refractory DLBCL with the agent Hu5F9-G4 (Magrolimab) and rituximab. Among the 15 patients treated with DLBCL, the ORR was 6/15 (40%), with a CR rate of 5/15 (33%) [78]. Magrolimab’s development in blood cancer has since been discontinued due to adverse events. There are additional ongoing clinical trials targeting CD47 in DLBCL, including the CD47 antagonist evorpacept (clinicaltrials.gov, NCT05025800).

## 6. Conclusions

Over the last 8 years, we have seen more FDA approvals for relapsed/refractory DLBCL than in the prior 25 years. These contributions are made possible largely due to a stronger understanding of molecular signaling pathways and immune-based therapies. While we have come a long way in regard to our understanding of signaling and targeted treatments, there is still a lot of room to improve outcomes for patients with relapsed/refractory DLBCL. Despite multiple new therapeutics in the relapsed/refractory setting (Table 1), there has been no OS benefit to any regimen when compared to standard R-CHOP in the front-line setting—cure rates remain stagnant around 60–70% [3] (Table 2). However, there is room for optimism as extracellular targets and T-cell-directing therapies continue to show promise. Subsequently, combining extracellular targets with more pathway-specific therapies (e.g., ViPOR, brentuximab vedotin + lenalidomide, etc.) will likely continue to improve outcomes. As discussed, our understanding of the BCR signaling, TLR pathways, the PI3K-AKT-mTOR axis, and extracellular targets have resulted in multiple new FDA-approved therapies. Further understanding of these pathways will allow us to tailor therapy towards specific genetic subtypes in DLBCL and enable us to achieve curative therapy in this high-risk patient population.

## Figures and Tables

**Table 1:** Novel agents utilized in relapsed/refractory DLBCL.

Drug	Mechanism of Action (MOA)	ORR in Relapsed/Refractory DLBCL
Ibrutinib [23]	BTK inhibitor	20/80 (25%) —ABC DLBCL 14/38 (37%) —GCB DLBCL 1/20 (5%)
Fostamatinib [30]	SYK inhibitor	2/68 (3%)
Entospletinib [31]	SYK inhibitor	0/43 (0%)
Enzastaurin [32]	PKCβ inhibitor	8/55 (15%) freedom from progression following 4 cycles
Lenalidomide [39]	NF-κβ inhibitor/CELMoD	30/108 (28%)
Bortezomib + Gemcitabine [79]	Down-regulates NF-κβ	1/16 (6%)
Copanlisib [47]	PI3K inhibitor	13/67 (19%)
MK2206 [80]	AKT inhibitor	0/9 (0%)
Everolimus + Rituximab [48]	mTOR inhibitor	9/24 (38%)
Venotoclax [52]	BCL2 inhibitor	6/34 (18%)
Selinexor [5] *	XPO1 inhibitor	36/127 (28%)
Glofitamab [81] *	CD20XCD3 bispecific antibody	54/107 (50%)
Epcoritamab [13] *	CD20XCD3 bispecific antibody	15/22 (68%)
Tafasitamab + Lenalidomide [6] *	anti-CD19	48/80 (60%)
Loncastuximab tesirine [7] *	CD19 antibody–drug conjugate	70/145 (48%)
Blinatumomab [63]	CD19/CD3 bispecific antibody	15/41 (37%)
Axicabtagene ciloleucel [10] *	CD-19 CAR-T cell	83/101 (82%)
Tisagenlecleucel [11] *	CD-19 CAR T-cell	48/93 (52%)
Lisocabtagene Maraleucel [12] *	CD-19 CAR T-cell	186/256 (73%)
Polatuzumab vedotin + bendamustine/rituximab [4] *	CD79 antibody–drug conjugate	60/106 (57%)
Zilovertamab vedotin [82]	ROR-1 antibody–drug conjugate	3/5 (6%)
Brentuximab-vedotin (BV) [70]	CD30 antibody–drug conjugate	21/48 (44%)
BV + Lenalidomide + Rituximab [8] *	CD30 antibody–drug conjugate with NF-κβ inhibitor	71/112 (64%)
Nivolumab [76]	PD-1 inhibitor	3/87 (3%)
Magrolimab + Rituximab [78]	CD47 inhibitor	6/15 (40%)

* FDA-approved for relapsed/refractory DLBCL.

**Table 2:** First-line trials in DLBCL.

First-Line Trial	Comparison	Results	
Pola-R-CHP [2]	Standard R-CHOP	2-year PFS 76.7% vs. 70.2%. No OS benefit	
Obin-CHOP [83]	Standard R-CHOP	No PFS or OS benefit	
R2-CHOP [84]	Standard R-CHOP	No PFS or OS benefit	- included patients with ABC-DLBCL
Ibrutinib-R-CHOP [85]	Standard R-CHOP	Worsened EFS, PFS, and OS	- included patients with non-GCB DLBCL - patients younger than 60 showed improved EFS, PFS, and OS
Bortezomib-R-CHP [86]	Standard R-CHOP	No difference in PFS or OS	
Venetoclax + R-CHOP [87]	Standard R-CHOP in GOYA trial [83]	Improved PFS (HR 0.61) No OS benefit	- increased hematologic grade 3/4 adverse events
R-Chemo followed by 1 yr of everolimus [88]	R-chemo followed by 1 yr of placebo	No difference in EFS	

Abbreviations: Pola-R-CHP: Polatuzumab vedotin + Rituximab + Cyclophosphamide + Doxorubicin + Prednisone. Obin-CHOP: Obinutuzumab + Cyclophosphamide + Doxorubicin + Vincristine + Prednisone. R2-CHOP: Lenalidomide + Rituximab + Cyclophosphamide + Doxorubicin + Vincristine + Prednisone. R-CHOP: Rituximab + Cyclophosphamide + Doxorubicin + Vincristine + Prednisone. Bortezomib-R-CHP: Bortezomib + Rituximab + Cyclophosphamide + Doxorubicin + Prednisone.
